# Mortality in individuals with intellectual disabilities in Finland

**DOI:** 10.1002/brb3.431

**Published:** 2016-01-24

**Authors:** Maria Arvio, Tommi Salokivi, Aila Tiitinen, Leena Haataja

**Affiliations:** ^1^ Department of NeurologyJoint Authority for Päijät‐Häme Social and Health CareLahtiFinland; ^2^ Support and Expert Center for Persons with Intellectual DisabilityKTOPaimioFinland; ^3^ Department of Obstetrics and GynaecologyHelsinki University Central Hospital and University of HelsinkiHelsinkiFinland; ^4^ Department of Child NeurologyTurku University Hospital and University of TurkuTurkuFinland

**Keywords:** Gender, intellectual disability, mortality, standardized mortality ratios

## Abstract

**Objectives:**

This study aimed at ascertaining the standardized mortality ratios (SMR) for those with an intellectual disability (ID) in Finland.

**Materials and Methods:**

We used the statistical database of the national insurance institution of Finland and Statistics Finland's mean population figures. We determined the number of individuals who received benefits (disability allowance, disability pension, or care allowance for pensioners) due to an ID diagnosis and the number of those whose benefit had been terminated due to death during the years 1996–2011.

**Results:**

SMR for females with a mild ID (IQ 50–69) was 2.8 (95% CI: 2.60–3.01) and for males 2.0 (95% CI: 1.88–2.14), and for females with a severe ID (IQ <50) 5.2 (95% CI: 4.99–5.50) and for males 2.6 (95% CI: 2.48–2.72).

**Conclusion:**

This significant difference in the SMR figures between males and females with ID warrants further research.

## Introduction

Those with an intellectual disability (ID) form a heterogenic group. IDs can be classified into subgroups based on their etiology (genetic, acquired or multifactorial), severity, and comorbidities. The most common genetic cause for ID is Down syndrome; the most frequent acquired condition is cerebral palsy‐associated ID, and the most common multifactorial condition is autism spectrum‐related ID syndrome (Arvio et al. 2014). Severe ID almost always associates with such other impairments as speech abnormalities, epilepsy, psychiatric disorders and movement disorder. Many with an ID deteriorate over time due to their underlying neurological conditions which may be progressive in nature (Arvio and Sillanpää 2003).

Worldwide, those with an ID, form the largest single disability group. In western countries approximately 0.7% of the population meets the diagnostic criteria of ID (Leonard and Wen 2002; Westerinen et al. 2007; Søndenaa et al. 2010). The proportion of males with an ID is greater than that of females (Westerinen et al. 2007; Yen et al. 2013). Several reasons explain the higher incidence among males. First, their more frequent autism spectrum disorders, and secondly, the fact that X‐chromosomal disorders such as Fragile‐X syndrome present with more severe cognitive symptoms in males. And finally, boys are more likely to experience traumatic brain injury. In general, no gender difference exists in ID severity (Memisevic and Sinanovic 2014). The age distribution of the ID population is skewed toward younger age groups (Yen et al. 2013).

In Finland, child neurological teams in public central hospitals are responsible for ID diagnostics, after which the individual receives a referral to one of the 16 regional state‐supported special care district's out‐patient clinics for a treatment, rehabilitation, and service plan. The main criteria for ID have remained unchanged for decades. These include an IQ below 70 in age‐appropriate psychological tests, age‐inappropriate adaptive skills, and clinical manifestation during the developmental period (WHO, 1996).

In Finland, the national social insurance institution (KELA) is primarily responsible for supporting those over the age of 15 with an ID by providing each with disability pension and care allowance. Furthermore, the parents of children and adolescents (under age 16) with an ID receive a disability allowance. ICD‐10 was adopted in Finland in 1996, and KELA uses its codes to classify beneficiaries. Therefore, the Finnish population registers provide a unique database for a more detailed examination of mortality rates. This study aimed to ascertain the standardized mortality ratios (SMR) for people with an ID in Finland.

## Methods

KELA's statistical database (www.kela.fi/tilastot) provided us with the number of individuals who received benefits (disability allowance, disability pension or care allowance for pensioners) due to an ID diagnosis (ICD 10: F70–79) during the years 1996–2011. The Finnish Population Register Centre delivers data of citizens’ deaths to KELA; the number of whose benefit had been terminated due to death (observed deaths) during this time period came also from KELA. The expected number of deaths was calculated on the basis of gender‐, 5‐year age groups, and calendar‐period‐specific mortality rates in the Finnish population (www.tilastokeskus.fi). The SMR was calculated as the ratio of observed and expected number of deaths. Ninety‐five percent confidence intervals (95% CI) were computed by assuming that the observed number of deaths followed a Poisson distribution.

## Results

**Figure 1:**
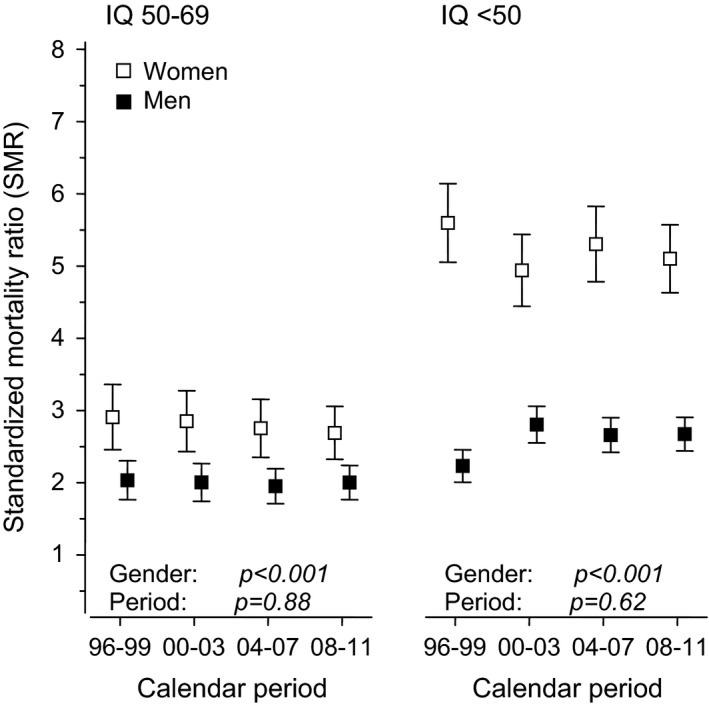
The standardized mortality rate (95% confidence intervals) of women and men with a mild (IQ 50–69) and a severe (Q < 50) intellectual disability (ID) during 1996–2011.

The SMR of all subjects with a mild ID (IQ 69–50) and a severe ID (IQ <50) during 1996–2011 according chronological age are presented in Table 1. SMR for females with a mild ID was 2.80 (95% CI: 2.60–3.01) and for males 2.01 (95% CI: 1.88–2.14) (*P* < 0.001), and for females with a severe ID 5.24 (95% CI: 4.99–5.50) and for males 2.59 (95% CI: 2.48–2.72) (*P* < 0.001) (Table 2). See the figure showing SMRs in calendar‐year periods according to gender and level of ID. Thorough the observation period, the considerable difference between genders in mortality rates in both ID levels remained stable (Fig. 1).

**Table 1:** The standardized mortality rate (SMR) of subjects with a mild (IQ 50–69) and a severe (Q < 50) intellectual disability (ID) during 1996–2011 according age

	Person years	Number of death		SMR (95% CI)^a^
		Observed	Expected	
Mild ID	151835	1698	177.0	2.28 (2.18 to 2.39)
0–14	12735	8	1.9	4.21 (1.82 to 8.30)
15–29	24006	41	17.5	2.34 (1.68 to 3.18)
30–44	38841	163	58.9	2.77 (2.36 to 3.23)
45–59	54562	693	266.8	2.60 (2.41 to 2.80)
≥60	21691	793	398.9	1.99 (1.85 to 2.13)
Severe ID	227152	3473	1019.1	3.41 (3.30 to 3.52)
0–14	33211	110	8.3	13.26 (10.90 to)
15–29	46850	279	31.8	8.77 (7.77 to 9.87)
30–44	57019	525	87.6	5.99 (5.49 to 5.53)
45–59	63941	1369	317.0	4.32 (4.09 to 4.55)
≥60	26131	1190	574.3	2.07 (1.96 to 2.19)

a Standardized mortality rate.

**Table 2:** The standardized mortality rate (SMR) of women and men with a mild (IQ 50–69) and a severe (Q < 50) intellectual disability (ID) during 1996–2011 according age

	Women			Men		
	Number of death		SMR (95% CI)^a^	Number of death		SMR (95% CI)^a^
	Observed	Expected		Observed	Expected	
Mild ID	724	485.3	2.80 (2.60 to 3.01)***	974	258.7	2.01 (1.88 to 2.14)
0–14	4	0.6	6.33 (1.72 to 16.20)	4	1.3	3.15 (0.86 to 8.08)
15–29	14	3.3	4.28 (2.34 to 7.18)*	27	14.2	1.90 (1.25 to 2.76)
30–44	60	14.3	4.20 (3.21 to 5.41)***	103	44.6	2.31 (1.89 to 2.80)
45–59	272	78.7	3.45 (3.06 to 3.89)***	421	188.1	2.34 (2.03 to 2.46)
≥60	374	161.8	2.31 (2.08 to 2.56)***	419	237.1	1.77 (1.60 to 1.94)
Severe ID	1641	313.2	5.24 (4.99 to 5.50)***	1832	705.9	2.59 (2.48 to 2.72)
0–14	68	3.2	21.35 (16.58 to 27.07)***	42	5.1	8.22 (5.92 to 11.11)
15–29	130	6.4	20.47 (17.10 to 24.31)***	149	25.5	5.85 (4.95 to 6.87)
30–44	249	21.3	11.71 (10.30 to 13.26)***	276	66.4	4.16 (3.68 to 4.68)
45–59	652	80.3	8.12 (7.51 to 8.77)***	717	236.7	3.03 (2.81 to 3.26)
≥60	549	202.1	2.68 (2.46 to 2.92)***	648	174.1	1.74 (1.61 to 1.88)

a Standardized mortality rate.

*P < 0.05, **P < 0.01, ***P < 0.001 between gender.

## Discussion

Our research confirms a difference in mortality risk between genders in Finland. In western countries men show higher mortality rate than women (http://www.who.int/nmh/publications/ncd_report_chapter1.pdf). Females with a severe ID had a fivefold higher SMR than Finnish females in general while the corresponding difference among males was twofold. The gender difference among those with a mild ID was smaller but also statistically significant. The corresponding SMR figures in non‐Finnish studies representing various ID‐related disability groups among females range from 7 to 17 and among males from 2 to 9 (Tyrer et al. 2007; Mouridsen et al. 2008; Gillberg et al. 2010; Woolfenden et al. 2012).

The strength of our data lies in its being registry‐based and consisting of all the individuals living in Finland who during 1996–2011 received a disability pension, disability allowance or care allowance (refundable drug benefits were not included). In some countries, individuals can choose between pension and full care without finances; this not the case in Finland. We assume that few citizens exist or have existed who have an ID and are sufficiently wealthy to live their entire lives without a disability benefit. Furthermore, anyone who earns a living hardly meets the criteria of ID. We therefore assume that the coverage of our study material is convincing.

The weakness of our data is the lack of death causes which have not been nationally registered.

In Finland alcoholism is the most common cause of death in males and the second most common cause in females under the age of 65 (www.terveyskirjasto.f/xmedia/ldk/). An ID, in itself, almost always makes substance addiction (with the exception of psychopharmacy) impossible, because as those with an ID generally live under supervision and are unable to obtain addictive substances by themselves. Breast cancer is the most common cause of death in females under the age of 65 (www.terveyskirjasto.f/xmedia/ldk/); an ID is not a known risk factor for breast cancer, although being childless is (Raitasuo et al. 1996; Patja et al. 2001; Tyrer and McGrother 2009). Several studies have shown that cardiovascular disease, the second most common cause of death for Finnish males and the fifth for Finnish females (www.terveyskirjasto.f/xmedia/ldk/), is the most common cause of death in those, specifically females, with an ID (Raitasuo et al. 1996; Patja et al. 2001). This indicates that the most important cause of the mortality difference between genders may be cardiovascular mortality.

The SMR difference between genders challenges us to further study of why female gender is a risk factor for those with ID suffering premature death, although being male is a risk factor for ID.

## Conflict of Interest

None of the authors has any conflict of interest to disclose.
